# Pigsties near dwellings as a potential risk factor for the prevalence of Japanese encephalitis virus in adult in Shanxi, China

**DOI:** 10.1186/s40249-017-0312-4

**Published:** 2017-06-08

**Authors:** Xiaojie Ren, Shihong Fu, Peifang Dai, Huanyu Wang, Yuanyuan Li, Xiaolong Li, Wenwen Lei, Xiaoyan Gao, Ying He, Zhi Lv, Jingxia Cheng, Guiqin Wang, Guodong Liang

**Affiliations:** 1 Department of Immunology and Microbiology, Shanxi Medical University, Taiyuan, 030001 China; 2 State Key Laboratory of Infectious Disease Prevention and Control, National Institute for Viral Disease Control and Prevention, Chinese Center for Disease Control and Prevention, Beijing, 102206 China; 3 Collaborative Innovation Center for Diagnosis and Treatment of Infectious Diseases, Hangzhou, 310058 China; 4 Shanxi Center for Disease Control and Prevention, Taiyuan, 030001 China

**Keywords:** Adult Japanese encephalitis, Epidemic disease, Mosquito-borne arbovirus, Japanese encephalitis virus

## Abstract

**Background:**

The increasing trend of adult cases of Japanese encephalitis (JE) in China, particularly in northern China, has become an important public health issue. We conducted an epidemiological investigation in the south of Shanxi Province to examine the relationships between mosquitoes, Japanese encephalitis virus (JEV), and adult JE cases.

**Methods:**

Mosquito specimens were collected from the courtyards of farmers’ households and pig farms in Shanxi Province. Mosquitoes were pooled, homogenized, and centrifuged. Reverse transcription-polymerase chain reaction (RT-PCR) was used to detect mosquito-borne arbovirus genes in homogenates. Specimens positive for these genes were inoculated into the baby hamster kidney cell line (BHK-21) to isolate virus. Minimum infection rate was calculated and phylogenetic analyses were performed.

**Results:**

A total of 7 943 mosquitoes belonging to six species in four genera were collected; *Culex tritaeniorhynchus* accounted for 73.08% (5 805/7 943), *C. pipiens pallens* for 24.75% (1 966/7 943), and the remaining 3% (104/ 7943) consisted of *Anopheles sinensis*, *Aedes vexans*, *Ae. dorsalis*, and *Armigeres subalbatus*. Sixteen pools were positive for JEV based on RT-PCR using JEV pre-membrane gene nested primers. Phylogenetic analyses showed that all JEVs belonged to genotype I; two pools were positive using Getah Virus (GETV) gene primers. In addition, one JEV strain (SXYC1523) was isolated from *C. pipiens pallens* specimens. These results indicate that the minimum infection rate of JEV in mosquito specimens collected from the courtyards of farmers’ households with pigsties was 7.39/1 000; the rate for pig farms was 2.68/1 000; and the rate for farmers’ courtyards without pigsties was zero.

**Conclusions:**

The high-prevalence regions of adult JE investigated in this study are still the natural epidemic focus of JEV. Having pigsties near dwellings is a potential risk factor contributing to the prevalence of adult JE. To prevent the occurrence of local adult JE cases, a recommendation was raised that, besides continuing to implement the Expanded Program on Immunization for children, the government should urge local farmers to cease raising pigs in their own courtyards to reduce the probability of infection with JEV.

**Electronic supplementary material:**

The online version of this article (doi:10.1186/s40249-017-0312-4) contains supplementary material, which is available to authorized users.

## Multilingual abstracts

Please see Additional file 1 for translations of the abstract into the six official working languages of the United Nations.

## Background

Japanese encephalitis (JE) is a central nervous system disease caused by Japanese encephalitis virus (JEV), which has severe symptoms and a fatality rate of 30%. About 35% of survivors have permanent neurological or psychiatric sequelae [1, 2]. JEV is transmitted by mosquitoes, among which *Culex tritaeniorhynchus* is the most important vector. Pigs and migratory birds are primary amplification hosts [1–3]. JE is mainly epidemic in developing countries in Asia such as China, India, Thailand, Vietnam, Myanmar, Laos, and Indonesia. It is also the most important form of viral encephalitis in these regions [3, 4]. JE mainly occurs in children up to 14 years old [1–4], but adult cases have been reported in recent years. In 2006, 66 cases of JE were reported with 22 deaths in Shanxi Province, China, among which only 1 patient was 4 years old and more than 86% were over 30 years old [5]. In recent years, the number of adult cases has exceeded pediatric cases in some endemic areas in India [6]. In addition, 129 cases were reported in South Korea during 2010–2015, among which patients older than 40 years accounted for 61.2% [7]. Therefore, the epidemic of adult JE in local regions has become a new public health issue.

China has the highest prevalence rates of JE, accounting for nearly 50% of the total number of cases reported around the world annually [4]. In 2008, China has included JE vaccination in the Expanded Program on Immunization (EPI), and children ≤15 years old in JE-endemic areas can be inoculated with JE vaccine at no cost; this has greatly reduced the incidence of JE in children [8–10]. However, the incidence of adult cases in some provinces of China is higher than the national average, and the increased proportion of cases in adults is much higher than that in children [10]. The increase in adult JE cases, particularly in patients over 40 years old, has gradually become the driving factor for the high national incidence of JE from 2004 to 2014 in China. Previous studies have shown that the number of JE cases in the ≤15 years old group decreased by 17% in 2013, while that in people >40 years old increased by 394.16% compared to 2012 [10]. There are six high-prevalence provinces for adult JE (Shanxi, Shandong, Henan, Hebei, Shaanxi, and Gansu), all of them located in north of the Yangtze River (30°N–35°N and 110°E–130°E). Spatial cluster analyses have suggested that the distribution of adult cases in the south of Shanxi Province have demonstrated spatial clusters in years with high JE prevalence rates. Thus, the high incidence of adult JE in the southern region of Shanxi Province has become a heavy burden on local public health [10].

**Fig. 1:**
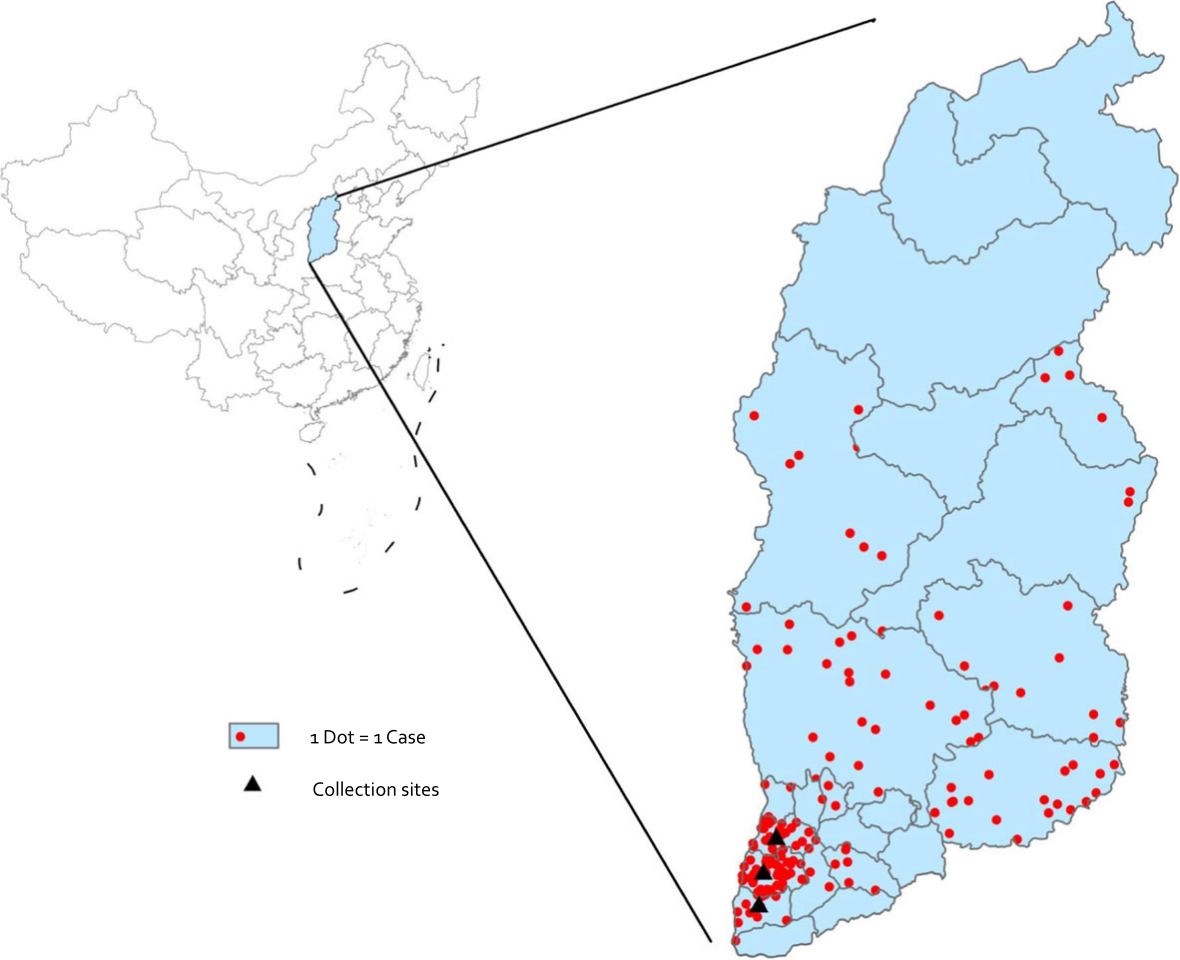
Geographical distribution of adult JE cases in Shanxi Province from 2009 to 2014, and the collection sites of mosquito specimens in this study. The triangles represent Wanrong, Linyi, and Yongji from top to bottom in the figure, respectively

A total of 253 JE cases were reported in Shanxi province from 2009 to 2014, among which adult JE cases (over 40 years old) accounted for 83% (210/253). The adult cases were mainly distributed in Linyi, Yongji, and Wanrong counties, accounting for 35.7% (75/210) of the total (Fig. 1). Therefore, we conducted an investigation in these three counties to understand the relationships between local mosquito vectors, JEV, and local adult JE cases.

## Methods

### Cells

The baby hamster kidney cell line (BHK-21) was used for virus isolation. Cells were cultured with Dulbecco’s Modified Eagle’s Medium (DMEM) (Gibco, Grand Island, NY), 6% fetal bovine serum (FBS) (Gibco), 1% 100 U/ml penicillin and streptomycin (prepared by the Institute of Virology), and maintained at 37 °C under an atmosphere of 5% CO_2_ [11, 12].

### Mosquito collection

Previous papers showed that the mosquito density peaked from June to August and August has the highest mosquito density in the study area. The study area is on the east coast of the Yellow River. In summer, it is hot and rainy suitable for mosquito breeding, which is in June to August. Farmers grow wheat, corn, rice, cotton, potato, sorghum, millet, soybean, apple and so on. Vegetation is dominated by deciduous broad-leaf forest. JE cases peaked in June and August in Shanxi Province. So we collected mosquitoes from August 17 to 23, 2015 [5, 9]. The three counties are located between 34.8°N and 35.4°N, 110.3°E and 110.83°E (Fig. 1) in the Yellow River basin, which includes a large part of the Yellow River alluvial plain. Therefore, there are abundant rivers and lakes. Mosquito specimens were collected throughout this region, in villages with populations of about 800–1 000 people (200–300 households) per village. The distance between each village was more than 5 km. The investigation sites were divided into three categories, as follows.

1. Courtyards of farmers’ households with pigsties: there were not only houses for human habitation, but also pigsties for 5–10 pigs in the courtyard. The pigs were all raised in the courtyards, and there were no large-scale pig farms in these villages.
2. Courtyards of farmers’ households without pigsties: all pigs were intensively bred in pig farms 2 km from the village. Therefore, there were no pigs raised in the farmers’ courtyards in these villages.
3. Pig farms: the pig farms with intensive breeding of about 1 000 pigs were located 2 km from the villages. Residents in the village rented them according to the number of pigs they owned. Full-time personnel were responsible for the daily breeding and management of pigs on the farms.

Mosquitoes were collected with Ultraviolet light traps (Wuhan Lucky Star Environmental Protection Technology Co. Ltd., Hubei, China) and MT-1 CO_2_ mosquito traps (Beijing Detailong Science and Technology Development Co. Ltd., Beijing, China). The traps were set before sunset at 5:00 PM and mosquitoes were collected from them the following morning at 7:00 AM. The trapped mosquitoes were killed by freezing at –20 °C for 30 min. The specimens were placed on ice, and identified under a microscope for morphological classification. Male mosquitoes were excluded. Female mosquitoes were combined into different pools ≤120 specimens according to species, collection site, and collection time. The information was marked and registered. The specimens were stored in liquid nitrogen until they were examined in the laboratory [11, 12].

### Virus isolation

Pools of mosquito specimens were homogenized using a Mixer Mill Tissuelyser II (Qiagen, Hilden, Germany) at 25 times per second for 3 min with stainless steel beads (*r* = 3 mm) in 2 ml sterile plastic tubes containing 1.5 ml Eagle’s medium supplemented with 5% 100 U/ml penicillin and streptomycin, 1% 30 g/L glutamine, and 1% 75 g/L NaHCO_3_. Then the samples were centrifuged at 13 000 rpm, 4 °C, for 30 min.

Aliquots of 100 μl clarified homogenates were inoculated into 5.5 cm^2^ Nunc tubes (Nunc, Roskilde, Denmark) covered with a BHK cell monolayer containing 100 μl Eagle’s medium for 1 h at 37 °C under an atmosphere of 5% CO_2_. Then the medium was replaced with 2 ml fresh medium and the tubes were maintained at 37 °C under an atmosphere of 5% CO_2_. The cytopathic effect (CPE) was examined every 8 h for 5 days. Control BHK-21 cells were also examined at each stage. At 70% CPE, the samples were stored at –80 °C until identification. Those without a CPE were blindly passaged for three successive generations in the same way [11, 12].

### RT-PCR and molecular identification

RNA was extracted from 140 μl aliquots of clarified homogenates or virus culture stocks with a Viral RNA Mini Kit (QIAamp; Qiagen, Valencia, CA) in accordance with the manufacturer’s protocol. Then the viral RNA was used as the template to prepare cDNA with random primers (6-mer) (Takara, Otsu, Japan) using Ready-To-Go™ You-Prime FirstStrand Beads (GE Healthcare, Little Chalfont, Buckinghamshire, UK). The primers used for mosquito-borne virus gene detection are shown in Table 1 [12–15]. In this study, we detected not only JEV genes but also common arboviruses that had been discovered in local mosquito specimens. PCR was performed with GoTaq® Green Master Mix, 2× (Promega, Madison, WI) using a Mastercycler (Eppendorf, Hamburg, Germany) as follows: initial denaturation at 95 °C for 4 min followed by 35 cycles of denaturation at 95 °C for 30 s, annealing at 55 °C for 30 s, and extension at 72 °C for 1 min, with a final extension at 72 °C for 10 min. Amplified products were detected by 1% agarose gel electrophoresis and sequenced. BLAST searches of the nucleotide sequences obtained were conducted against GenBank to identify the types of virus genes in the specimens [11, 12].

**Table 1:** Primers used for identification in this study^a^

Primers	Sequence of primers (5′–3′)	Amplify region	Length of product ^(reference)^
Flavivius			
FU1	TACCACATGATGGGAAAGAGAGAGAA	NS5	310 [11]
CFD2	GTGTCCCAGCCGGCGGTGTCATCAGC		
Alphavirus			
M2W	YAGAGCDTTTTCGCAYSTRGCHW	NS1	434/310 [11]
cM3W	ACATRAANKGNGTNGTRTCRAANCCDAYCC		
M2W2	TGYCCNVTGMDNWSYVCNGARGAYCC		
Bunyaviruses			
BUP	ATGACTGAGTTGGAGTTTGATGTCGC	S	251 [13]
BDW	TGTTCCTGTTGCCAGGAAAAT		
BAV S12 gene primers			
BAV-12-854-S	AAATTGATAGYGYTTGCGTAAGAC	S12	850 [11]
BAV-12-B2-R	GTTCTAAATTGGATACGGCGTGC		
LNV S12 gene primers			
LNV12s1	CACTGGCTCCGGCTGTAGTAACAG	S12	435 [14]
LNV12r1	CTGTTCGGATCATCTGGAATTTGA		
GETV 5′UTR and NS1 gene primers			
F1	ATGGCGGACGTGTGACATCAC	5′UTR,NS1	930 [15]
R1	GTAACCTTCGCATGACACCACC		
JEV C/PrM gene primers			
JE-251 F	CGTTCTTCAAGTTTACAGCATTAGC	C/PrM	674/492 [5]
JE-925R	CCYRTGTTYCTGCCAAGCATCCAMCC		
JE-743R	CGYTTGGAATGYCTRGTCCG		

F, Forward primer; R, Reverse primer; M, C/A; W, A/T; Y, C/T; K, G/T; R, G/A; V, G/A/C; D, T/A/G; BAV, Bannan virus; LNV, Liaoning virus; GETV, Getach virus; JEV, Japanese encephalitis virus

^a^ The primers used to amplify the complete open reading frame (ORF) nucleotide sequence and envelope gene of the viral genomic RNA were all from a previous study [23]

### Minimum infection rate

Minimum infection rate (MIR) was calculated as the (number of pools positive for JEV/total number of specimens tested) × 1 000, assuming that every positive pool contained only one infected mosquito. This was calculated for each mosquito species and each mosquito collection site during the study [16].

### Phylogenetic analysis

Seqman software (DNAStar, Madison, WI) was used for sequence splicing and quality analysis of the original nucleotide sequence. Additional JEV sequences were downloaded from GenBank. The JEV strains used in this study with source and region of isolation are listed in Table 2. BioEdit software (version 7.0.5.3; Thomas) was used for multiple alignment by ClustalW.MegAlin software (DNAStar) was used to convert nucleotide sequences into amino acid sequences and to separately compare nucleotide and amino acid sequence identities.

**Table 2:** Strains of Japanese encephalitis virus used in this study

Strain	Genotype	Year	Country and region	Source	GenBank accession No.	
					E gene	Complete gene
SXYC1523*	I	2015	Shanxi,China	*Culex pipiens*	KY078829	KY078829
SXYC1546*	I	2015	Shanxi,China	*C. tritaeniorhynchus*	KY078827	
SXYC1548*	I	2015	Shanxi,China	*C. tritaeniorhynchus*	KY078828	
Ishikawa	I	1994	Ishikawa, Japan	Swine mononuclear cells	AB051292	AB051292
JEV/sw/Mie/40/2004	I	2004	Japan	Pig serum	AB241118	AB241118
12-YJ033	I	2012	Shanxi,China	*C. tritaeniorhynchus*	KP216590	
SX09S-01	I	2008	Shanxi,China	Pig brain	HQ893545	HQ893545
12-LY039	I	2012	Shanxi,China	*C. pipiens*	KP216598	
12-YJ022	I	2012	Shanxi,China	*C. tritaeniorhynchus*	KP216587	
XJ69	I	2007	China	*C. pipiens pallens*	EU880214	EU880214
SH03-130	I	2003	Shanghai, China	*C. tritaeniorhynchus*	DQ404104	
KV1899	I	1999	Korea	Pig serum	AY316157	AY316357
YN79-Bao83	I	1979	Yunan, China	*C. tritaeniorhynchus*	DQ404128	
YN-Xiang JE	I	IU	Yunan, China	Human blood	DQ404135	
LN02-102	I	2002	Liaoning, China	*C. modestus*	DQ404085	
SH03-105	I	2003	Shanghai, China	*C. tritaeniorhynchus*	DQ404097	
HN06-21	I	2006	Henan, China	*Culex*	JN381830	
HN06-26	I	2006	Henan, China	*Culex*	JN381837	
SC04-12	I	2004	Sichuan, China	*Culex*	DQ404090	
GZ56	I	2008	Guizhou, China	Cerebrospinal fluid	HM366552	HM366552
JEV/sw/Mie/41/2002	I	2002	Mie, Japan	Swine serum	AB241119	AB241119
K94P05	I	1994	South Korea	*C. tritaeniorhynchus*	AF045551	AF045551
XJP613	I	2007	China	*C. tritaeniorhynchus*	EU693899	EU693899
FU	II	1995	Australia	Human sreum	AF217620	AF217620
SA14	III	1954	China	Mosquito	U14163	U14163
SA14-14-2	III	IU	China	Vaccine	AF315119	AF315119
P3	III	1949	Beijing, China	Human brain	U47032	U47032
Nakayama-RFVL	III	1935	Nakayama, Japan	Human brain	S75726	
GZ04-36	III	2004	Guizhou, China	*Culex*	DQ404112	
HLJ02-134	III	2002	Heilongjiang, China	*Culicoides*	DQ404081	
FJ03-31	III	2003	Fujian, China	Human blood	DQ404117	
SH0601	III	2006	Shanghai, China	Pig	EF543861	EF543861
K87P39	III	1987	Korea	Mosquito	AY585242	AY585242
JaGAr01	III	1959	Japan,Gunma	*C. tritaeniorhynchus*	AF039076	AF039076
RP-9	III	1985	Taiwan,China	Mosquito	AF14161	AF14161
T1P1	III	1997	Taiwan,China	*Armigeres subalbatus*	AF254453	AF254453
Beijing-1	III	1949	Beijing, China	Human brain	L48961	L48961
Ling	III	1965	Taiwan,China	Mosquito	L78128	L78128
P20778	III	1958	India	Human brain	AF080251	AF08251
JKT6468	IV	1981	Indonesia,Flores	*C. tritaeniorhynchus*	AY184212	AY184212
Muar	V	1952	Malaysia	Human brain	HM596272	HM596272
XZ0934	V	2009	China	Mosquito	JF915894	JF915894
MVE		1951	Australia	Human brain	NC_000943	NC_000943

^*^Isolated from the study

Phylogenetic analyses were performed by the neighbor-joining (NJ) method using Mega software with 1000 bootstrap replicates. To generate rooted trees, Murray Valley encephalitis virus (MVE) was used as an outgroup in the JEV phylogenetic analysis [11, 12].

## Results

### Distribution of mosquitoes

A total of 7 943 mosquitoes were collected from Linyi, Yongji, and Wangrong counties, Shanxi Province, from 17 to 22 August 2015, and consisted of six species from four genera (Table 3); *Culex tritaeniorhynchus* accounted for 73.08% (5 805/7 943), *C. pipiens pallens* for 24.75% (1 966/7 943), and *Anopheles sinensis*, *Aedes vexans*, *Ae. dorsalis*, and *Armigers subalbatus* for about 3% (104/7 943). *C. tritaeniorhynchus* was the dominant species in all counties, accounting for 70.81% (1 994/2 816), 77.03% (2 505/3 252), and 69.65% (1 306/1 875) of specimens from Linyi, Yongji, and Wangrong counties, respectively.

**Table 3:** Mosquitoes collected in Shanxi, China, 2015

Mosquito species	Collection sites						Total	
	Linyi		Yongji		Wanrong			
	No.	%	No.	%	No.	%	No.	%
*Culex tritaeniorhynchus*	1 994	70.81	2 505	77.03	1 306	69.65	5 805	73.08
*C. pipiens pallens*	765	27.17	704	21.65	497	26.51	1 966	24.75
*Anopheles sinensis*	57	2.02	3	0.09	44	2.35	104	1.31
*Aedes vexans*	0	0	35	1.08	0	0	35	0.44
*Aedes dorsalis*	0	0	5	0.15	0	0	5	0.06
*Armigers subalbatus*	0	0	0	0	28	1.49	28	0.35
Total	2 816	100	3 252	100	1 875	100	7 943	100

### Molecular identification of mosquito-borne viruses

The mosquitoes were divided into 88 pools according to collection site, time, and species for homogenizing. RNA was extracted from 140 μl aliquots of clarified homogenates. The viral RNA was used as the template for RT-PCR using the seven mosquito-borne arbovirus primer sets listed in Table 1. The results are shown in Table 4. Among the 88 pools of mosquitoes, 16 were JEV-positive by RT-PCR amplification of the C/prM gene, among which 12 were *C. tritaeniorhynchus* and four were *C. pipiens pallens*. SXYC1546 and SXYC1548 specimens were JEV-positive by RT-PCR amplification of the JEV E gene. Sequence data for the E gene of SXYC1546 and SXYC1548 were deposited in GenBank. Among the 88 pools, 2 were positive for GETV using the 5′ UTR and NS1 gene primers. One SXYC1503 specimen (*C. tritaeniorhynchus*) was positive for both JEV and GETV at the same time. The collection site and mosquito species of positive specimens are listed in Table 4.

**Table 4:** Specimens positive for mosquito-borne virus genes in Shanxi, China, 2015 by RT-PCR amplifications

Collection site	Mosquito species	Sample title	viruses	No. Of each pool
The courtyards of farmer A’ households with pigsties	*Culex tritaeniorhynchus*	SXYC1537	JEV	100
The courtyards of farmer B’ households with pigsties	*C. tritaeniorhynchus*	SXYC1503	JEV/GETV	75
	*C. pipiens pallens*	SXYC1523^a^	JEV	20
	*C. tritaeniorhynchus*	SXYC1527	JEV	48
Pig farm A	*C. tritaeniorhynchus*	SXYC1542	JEV	100
	*C. tritaeniorhynchus*	SXYC1562	JEV	100
Pig farm B	*C. pipiens pallens*	SXYC1530	JEV	100
	*C. tritaeniorhynchus*	SXYC1546	JEV	100
	*C. tritaeniorhynchus*	SXYC1548	JEV	100
	*C. tritaeniorhynchus*	SXYC1549	JEV	100
	*C. tritaeniorhynchus*	SXYC1551	GETV	100
	*C. tritaeniorhynchus*	SXYC1553	JEV	100
	*C. tritaeniorhynchus*	SXYC1555	JEV	100
The courtyards of farmer C’ households with pigsties	*C. tritaeniorhynchus*	SXYC1570	JEV	100
	*C. tritaeniorhynchus*	SXYC1582	JEV	100
	*C. pipiens pallens*	SXYC1586	JEV	100
	*C. pipiens pallens*	SXYC1588	JEV	100

^a^Virus isolation obtained

1. Mosquitoes were collected from eight courtyards of farmers’ households (three with pigsties and five without pigsties) and two pig farms

2. The 16 pools of mosquito specimens positive for JEV were collected from the courtyards of three farmers’ households with pigsties (farmers A, B, and C) and two pig farms (pig farms A and B)

### Virus isolation and identification

**Fig. 2:**
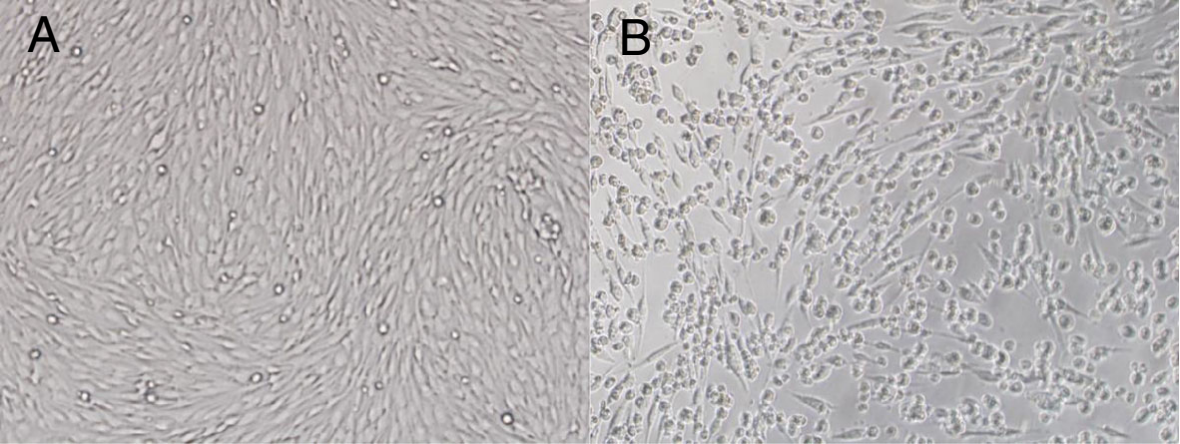
Phase-contrast photomicrographs of control and infected BHK-21 cells. **a** Control cells. **b** Cells 4 days after infection with SXYC1523

The clarified homogenates that were positive for JEV and GETV were inoculated onto BHK-21cells at a constant temperature, and CPE was observed under an optical microscope every 8 h. Among 17 pools of mosquitoes, only the SXYC1523 specimen isolated from *C. pipiens pallens* caused CPE in BHK-21 cells. Cells became round and shrank on day 3 after inoculation, CPE was up to 75% on day 4, and a large number of cells detached from the wall of the Nunc tube (Fig. 2). No obvious CPE was observed in other pools compared to control cells.

Viral RNA was extracted from cell culture supernatant of SXYC1523 and RT-PCR was conducted with arbovirus gene primers. The cell supernatant was positive for JEV. Then 16 overlapping primers were used to amplify the complete open reading frame (ORF) of the SXYC1523 strain. The sequence of the ORF has been deposited in GenBank.

### MIR of JEV in mosquitoes

Mosquito specimens were collected from the courtyards of eight farmers’ households and two pig farms in Linyi, Yongji, and Wangrong counties. Of 45 pools of mosquito specimens from the courtyards of three farmers’ households with pigsties (farmers A, B, and C) and two pig farms (pig farms A and B), 16 pools were positive for JEV in RT-PCR. The MIR of JEV from *Culex*, including *C. tritaeniorhynchus* and *C. pipiens pallens*, collected from three farmers’ households with pigsties was 7.39/1 000, and that from *Culex* collected from the two pig farms was 2.68/1 000. Thus, the virus carrier rate of JEV in mosquito specimens collected from the courtyards of farmers’ households with pigsties was as high or even higher than that from pig farms. Forty-three pools of mosquitoes collected from the courtyards of five farmers’ households without pigsties were negative for JEV (Table 5).

**Table 5:** Minimum infection rate (MIR) of JEV in mosquitoes in this study

Collection sites	Mosquito species	No. Individuals	No.pools	No. Positive Pools	MIR (/1000)
The courtyards of farmers’ households with pigsties^a^	*Culex tritaeniorhynchus*	723	8	5	6.92
	*C. pipiens pallens*	360	5	3	8.3
	Subtotal	1 083	13	8	7.39
Pig farm^b^	*C.tritaeniorhynchus*	2 433	26	7	2.88
	*C. pipiens pallens*	552	6	1	1.81
	Subtotal	2 985	32	8	2.68
The courtyards of farmers’ households without pigsties^c^	*C. tritaeniorhynchus*	2 649	33	0	0
	*C. pipiens pallens*	1 054	10	0	0
	Subtotal	3 703	43	0	0

^a^Courtyards of three farmers’ households with pigsties (farmers A, B, and C shown in Table 4)

^b^Two pig farms (pig farms A and B in Table 4)

^c^Courtyards of five farmers’ households without pigsties

### Molecular characterization of mosquito-borne viruses

#### Phylogenetic analysis

**Fig. 3:**
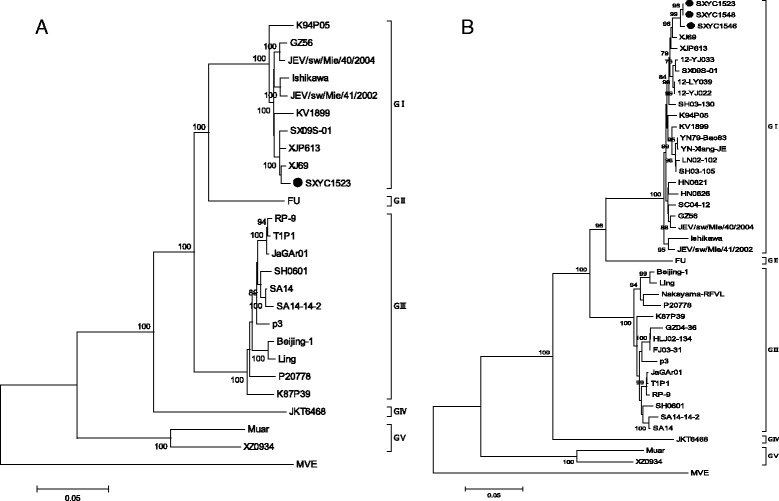
Phylogenetic analysis of JEV isolates. **a** Phylogenetic analysis based on ORF sequencing. **b** Phylogenetic analysis based on E gene sequencing. Scale bars indicate the number of nucleotide substitutions per site

To understand the molecular genetic characteristics of the JEV isolates obtained in the present study, we selected 39 JEV strains covering genotypes I–V isolated from different countries and different species of mosquitoes from GenBank to establish phylogenetic trees based on the E gene and ORF sequence together with the new isolates in this study. JEV was divided into five genotypes, and SXYC1523 isolated from *C. pipiens pallens* in Shanxi was located in the branch of genotype I (Fig. 3a). In phylogenetic analyses based on the E gene (Fig. 3b), SXYC1523, SXYC1546, and SXYC1548 derived from mosquitoes in Shanxi Province in 2015 were all located in the branch of genotype I.

### JEV identity and variation in amino acid sequences

The levels of nucleotide and amino acid sequence identity of the JEV E gene were 99.5–100% and 100%, respectively, in three strains (SXYC1523, SXYC1546, SXYC1548). Comparison of the nucleotide and amino acid sequences of the E gene between SXYC1523 strain and 39 other strains used in phylogenetic analyses indicated a nucleotide identity ranging from 72.8% (XZ0934) to 98.7% (XJ69) and an amino acid identity ranging from 90.6% (XZ0934) to 100%. The nucleotide sequence identity of the E gene between SXYC1523 with genotype I JEV ranged from 96.3% (Ishikawa) to 98.7% (XJ69), and the amino acid sequence identity ranged from 98% (Ishikawa) to 100%. Amino acid sequence identity of the E protein between the SXYC1523 strain and local JE strains (12-YJ033, 12-LY039, 12-TJ022) isolated in 2012 was 100%.

The E protein is a major structural protein of JEV and is closely related to viral virulence. To analyze the key amino acids, we compared the E protein of strains isolated in this study (SXYC1523, SXYC1546, SXYC1548) to SA14-14-2, an attenuated vaccine strain, and other virulent strains (Table 6). The results suggested that eight key amino acid residues were not different in these three strains derived from mosquitoes collected in the areas with a high incidence of adult JE in this study, compared to JEV strains isolated from mosquitoes, porcine serum, or specimens from patients with encephalitis, regardless of genotype. These results suggest that the virulence of JEV circulating in these regions in 2015 has not changed.

**Table 6:** Comparison of key amino acid residues of the E protein related to neurovirulence of JEV^a^

Strain	E107	E138	E176	E177	E264	E279	E315	E439
SA-14-14-2 (GIII)	Phe(F)	Lys(K)	Val(V)	Ala(A)	His(H)	Met(M)	Val(V)	Arg(R)
SXYC1523^b^(GI)	Leu(L)	Glu(E)	Ile(I)	Thr(T)	Gln(Q)	Lys(K)	Ala(A)	Lys(K)
SXYC1546^b^(GI)	Leu(L)	Glu(E)	Ile(I)	Thr(T)	Gln(Q)	Lys(K)	Ala(A)	Lys(K)
SXYC1548^b^(GI)	Leu(L)	Glu(E)	Ile(I)	Thr(T)	Gln(Q)	Lys(K)	Ala(A)	Lys(K)
SX09S-01(GI)	Leu(L)	Glu(E)	Ile(I)	Thr(T)	Gln(Q)	Lys(K)	Ala(A)	Lys(K)
12-YJ033(GI)	Leu(L)	Glu(E)	Ile(I)	Thr(T)	Gln(Q)	Lys(K)	Ala(A)	Lys(K)
GZ56(GI)	Leu(L)	Glu(E)	Ile(I)	Thr(T)	Gln(Q)	Lys(K)	Ala(A)	Lys(K)
FU(GII)	Leu(L)	Glu(E)	Ile(I)	Thr(T)	Gln(Q)	Lys(K)	Ala(A)	Lys(K)
Nakayama(GIII)	Leu(L)	Glu(E)	Ile(I)	Thr(T)	Gln(Q)	Lys(K)	Ala(A)	Lys(K)
P3 (GIII)	Leu(L)	Glu(E)	Ile(I)	Thr(T)	Gln(Q)	Lys(K)	Ala(A)	Lys(K)
JKT6468(GIV)	Leu(L)	Glu(E)	Ile(I)	Thr(T)	Gln(Q)	Lys(K)	Ala(A)	Lys(K)
Muar(GV)	Leu(L)	Glu(E)	Ile(I)	Thr(T)	Gln(Q)	Lys(K)	Ala(A)	Lys(K)
XZ0934(GV)	Leu(L)	Glu(E)	Ile(I)	Thr(T)	Gln(Q)	Lys(K)	Ala(A)	Lys(K)

^a^These eight aa residues of the E protein were shown to play a key role in neurovirulence. They are very different between the attenuated vaccine strain (SA14-14-2) and the virulent strains

^b^Isolated in Shanxi, 2015 in this study

## Discussion

JE is mainly endemic to Asia [1, 2, 4]. The scope of JE prevalence, however, has been gradually expanding in recent years, and JE has already spread to northwest Australia and Guam in the Pacific region, where it has become an emerging arboviral disease [17–19]. JEV is a mosquito-borne virus, and mosquitoes belonging to various genera, such as *Culex*, *Anopheles*, *Armigeres*, and *Aedes*, can all transmit it. Among these species, *Culex*, in particular *C. tritaeniorhynchus*, is the most important vector [19, 20]. The larvae of *C. tritaeniorhynchus* prefer to propagate in clean water, such as the water in rice fields, while the larvae of *C. pipiens pallens* generally propagate in sewage and the adults inhabit human dwellings. Therefore, it is easy for mosquitoes to propagate in rural areas with rich water resources, poor sanitation, and sewage [19, 20]. Pigs become infected with JEV via mosquito bites, and the virus is greatly amplified in pigs. This makes pigs, including both domestic and feral pigs, amplification hosts for local endemic JEV [19–21]. The infected pigs may also be hosts for further spread of JEV by mosquito bites. Therefore, a short distance between dwelling places and pigsties or the habitats of feral pigs will increase the probability of exposure to JEV. Populations living in environments with high mosquito density and surrounded by pigsties will be prone to JEV infection [22].

Our results suggest that the dominant mosquito specie in Linyi, Yongji, and Wanrong counties of Shanxi Province is still *C. tritaeniorhynchus*, and the endemic JEVs belong to genotype I, consistent with most parts of China and Asia [23]. The eight key amino acid residues determining the virulence of JEV isolates in this study have not changed compared to previous strains and local strains isolated in 2012, suggesting that local endemic JEV shows high neurovirulence [24]. These results suggest that the dominant mosquito species, genotypes, and virulence of JEV have not changed in Linyi, Yongji, and Wanrong, where the incidence of adult JE has been continuously high. Hence, these regions are still natural endemic foci of JEV with persist risk of infection.

In this study, we collected mosquito specimens from the courtyards of eight farmers’ households and two pig farms. There were pigsties in the courtyards of three farmers’ households but not in those of the other five households. Five to ten pigs were raised in the pigsties in the courtyards, and these pigsties were close to human houses. In addition, chickens, ducks, geese, and other domestic animals were also raised in the courtyards at the same time. Therefore, there was a great deal of stagnant water polluted by the feces of various animals in the living environment, which provided an appropriate environment for mosquitoes to propagate. Eight of thirteen pools of mosquito specimens collected from the courtyards of the above three farmers’ households with pigsties were positive for JEV based on RT-PCR amplification of the C/PrM gene, and the MIR was 7.39/1 000, higher than that (2.68/1 000) of mosquitoes collected from pig farms (Table 5). For the other five courtyards of the farmers’ households without pigsties, their pigs were all raised in pig farms far away from villages (over 2–5 km). Although large numbers of *C. tritaeniorhynchus* and *C. pipiens pallens* were present in the above five courtyards, JEV was not detected from these mosquitoes. Therefore, it is clear that whether the mosquitoes carried JEV was directly related to the location of pigsties in the courtyards (Table 5). The transmission cycle of JEV was blocked due to the lack of amplification hosts in the above five courtyards without pigsties. In contrast, the presence of pigs in the other three courtyards with pigsties completed the circle of JEV transmission as mosquito (virus)–pig–mosquito (virus), which made JEV more active and resulted in large numbers of mosquitoes carrying the virus. This concept is supported by another example from South Korea. During 2010–2015, South Korea reported 129 JE cases, some of which lived close to pigsties [7]. In conclusion, the presence of pigsties close to human dwellings provides an amplification host for JEV, which leads to JEV proliferation in local areas and increases the risk of human infection with JEV.

## Conclusion

A JE vaccine was included in the EPI in 2008 in China, and children can be inoculated with it free of charge; it has greatly reduced the incidence of JE cases among children in China [9, 22]. Adults were not inoculated with this vaccine in childhood (long before the implementation of EPI) and therefore are more susceptible to JEV infection [10]. In addition, the habit of farmers to raise pigs in their own courtyards increases the risk of infection with JEV. Therefore, in regions with high prevalence rates of adult JE, such as Linyi, Yongji, and Wanrong, it is necessary to implement JE vaccination and strengthen the management of local animal husbandry. Pigs should be raised intensively in pig farms far from human dwellings with implementation of modern management. Farmers should cease the practice of raising pigs in their own courtyards to reduce the risk of infection with JEV and further decrease the incidence of adult JE.

## Additional file

Additional file 1: Multilingual abstracts in the six official working languages of the United Nations. (PDF 633 kb)

## Abbreviations

BHK-21: Baby hamster kidney cell line
CPE: Cytopathic effect
DMEM: Dulbecco’s Modified Eagle’s Medium
EPI: The national Expanded Program of Immunization
FBS: Fetal bovine serum
GETV: Getah virus
JE: Japanese encephalitis
JEV: Japanese encephalitis virus
MIR: Minimum infection rate
MVE: Murray Valley encephalitis virus.
NJ: Neighbor-joining
ORF: Open reading frame
RT-PCR: Reverse transcription-polymerase chain reaction
