# Supplementary material for: Pigsties near dwellings as a potential risk factor for the prevalence of Japanese encephalitis virus in adult in Shanxi, China
Source: Infect Dis Poverty. 2017 Jun 8;6:100. doi: 10.1186/s40249-017-0312-4 (PMC5463306; doi:10.1186/s40249-017-0312-4)
Supplement: Additional file 1: — Multilingual abstracts in the six official working languages of the United Nations. (PDF 633 kb) [file 40249_2017_312_MOESM1_ESM.pdf]

Translation of the abstract into the six official working languages of the United Nations

وجود زرائب الخنازير بالقرب من المساكن كعامل خطر محتمل لانتشار التهاب الدماغ الياباني في البالغين في الصين

شياو جيه رن، شيهونج فو، بي آيفانج داي، هوانيو وانغ، يوان يوان لى، شياولونغ لي، وينوين لى، شياو يان قاو، يينغ هي، زهي ألفي، جينغشيا تشنغ، غويكين وانغ، قوه دونغ ليانغ

خلاصة

خلفية: لقد أصبحت ظاهرة ازدياد حالات التهاب الدماغ الياباني (JE) في البالغين في الصين، وخاصة شمال الصين، من قضايا الصحة العامة الهامة. قمنا بإجراء دراسة وبائية في جنوب مقاطعة شانشى لدراسة العلاقات بين البعوض وفيروس التهاب الدماغ الياباني (JEV)، وحالات التهاب الدماغ الياباني في البالغين.

المنهج: تم جمع عينات البعوض من باحات منازل المزارعين ومزارع الخنازير فى مقاطعة شانشى. كما تم تجميع البعوض، ومجانستها، ومعالجتها بالتنبيذ. وقد استخدم سلسلة تفاعل البلمرة - النسخ العكسي (PCR-RT) للكشف عن جينات فيروس أربوفيروس المنقول من قبل البعوض في الخليط المجانس. تم حقن العينات الإيجابية لهذه الجينات في خط الخلايا الكلوية في صغير الهامستر (21-BHK) من أجل عزل الفيروس. تم حساب الحد الأدنى لمعدل الإصابة، وإجراء تحاليل تطور السلالات.

النتائج: 7943 من البعوض تنتمي لستة أنواع ومن أربعة أجناس تم جمعها; *tritaeniorhynchus*‏*حيث مثل* 73.08% (7943/5805), *Culex pipiens pallens* for 24.75% (1966/7943), and the remaining 3% (104/7943) consisted of *Anopheles sinensis*, *Aedes vexans*, *Aedes dorsalis*, and *Armigeres suba*lbatus. أظهرت ستة عشرة بركة نتائج إيجابية لـ JEV استناداً إلى اختبار PCR-RT باستخدام جيف متداخلة الجينات الغشاء قبل الإشعال. وأظهرت تحليلات تطور السلالات أن جميع JEVs تنتمي إلى النوع الأول؛ وكانت اثنتان من البرك ايجابية باستخدام Getah فيروس (GETV) الاشعال الجينات. وبالإضافة إلى ذلك، تم عزل سلالة واحدة JEV سلالة (SXYC1523) من *C. النابصة pallens* عينات. تشير هذه النتائج إلى أن معدل الإصابة بالحد الأدنى من جيف في عينات البعوض التي تم جمعها من باحات منازل المزارعين المشتملو على زرائب خنازير كان 1000/7,39؛ وكان المعدل لمزارع الخنازير 000 1/2.68؛ وكان المعدل لأفنية المزارعين دون زرائب خنازير صفر.

الاستنتاجات: <bx/>مناطق معدل الانتشار العالي لمرض JE البالغين والتي تم دراستها في هذه الدراسة لا تزال مناطق انتشار JEV الطبيعية. وجود زرائب بالقرب من المساكن هو عامل خطر محتمل يسهم في انتشار التهاب الدماغ الياباني في البالغين. لمنع حدوث حالات التهاب الدماغ الياباني في البالغين محليا، تم رفع توصية بأنه، إلى جانب الاستمرار في تنفيذ برنامج التحصين الموسع للأطفال، يجب على الحكومة حث المزارعين المحليين على وقف تربية الخنازير في الساحات الخاصة للحد من احتمال الإصابة بفيروس JEV .

Translated from English version into Arabic by SAlkhodair, through

TRANSLATORS
WITHOUT BORDERS

农户家庭散养猪可能是中国成人乙脑发病的潜在危险因素

任晓杰，付士红，代培芳，王环宇，李元元，李晓龙，雷雯雯，高晓艳，何英，吕志，程璟侠，王桂琴，梁国栋

摘要

引言：自2006年以来，山西省临猗县，永济县和万荣县出现大量成人乙脑病例，成为当地重要

公共卫生问题。本文通过分析农户居住环境中养猪与否，环境中蚊虫携带乙脑病毒状况等，以了解农户散养猪的生活习惯与当地成人乙脑发病之间的关系。
方法：在调查地点采集蚊虫，经形态学分类鉴定，液氮保存运输到实验室备用。蚊虫标本按照种类和采集地等在实验室分批研磨离心处理。所有蚊虫研磨上清使用组织培养法开展病毒分离并使用分子生物学方法（RT-PCR）检测蚊虫研磨上清液中乙脑病毒等蚊传虫媒病毒。
结果：2015年8月在山西省临猗、永济、万荣县农户和猪场共采集到 4属6种7 943只蚊虫，其中三带喙库蚊为蚊虫总数73.08%（5 805/7 943），淡色库蚊为24.75%（ 1966/7 943）,其余3%（104/7 943）为中华按蚊，刺扰伊蚊，背点伊蚊和骚扰阿蚊。虫媒病毒基因检测（RT-PCR）到16批蚊虫标本为乙脑病毒基因阳性，2批为盖塔病毒阳性。进一步分析发现，庭院中散养猪的农户家和猪场采集的蚊虫标本均能检测到乙脑病毒，相反，不养猪的农户庭院中采集的蚊虫标本中未检测到乙脑病毒。
结论：本研究结果提示，本次调查的山西省临猗县，永济县和万荣县依然为乙脑病毒自然疫源地，而农户庭院中散养猪可能为乙脑高发的危险因素，因此杜绝农户庭院散养猪的生活习惯可以降低人群感染乙脑病毒机会，是降低乙脑发病率的重要措施之一。

Translated from English version into Chinese by Xiaojie Ren, modified by Guodong Liang.

Les exploitations porcines à proximité des habitations susceptibles d'être un facteur de risque pour la prévalence de l'encéphalite japonaise chez l'adulte en Chine

Xiaojie Ren, Shihong Fu, P Dai, Huanyu Wang, Yuanyuan Li, Xiaolong Li, Wenwen Lei, Xiaoyan Gao, Ying He, Zhi Lv, Jingxia Cheng, Guiqin Wang, Guodong Liang

Résumé
Contexte: L'augmentation des cas d'encéphalite japonaise (EJ) chez l'adulte en Chine est devenu un important problème de santé publique. en particulier dans le nord. Nous avons mené une enquête épidémiologique dans le sud de la province du Shanxi pour examiner les interactions entre les moustiques, le virus de l'encéphalite japonaise (VEJ) et les cas d'encéphalite japonaise (EJ) chez les adultes.
Procédés: Plusieurs espèces de moustiques ont été collectées dans les cours des agriculteurs locaux ainsi que dans les exploitations porcines dans la province du Shanxi. Les moustiques ont été rassemblés, homogénéisés et centrifugés. Une transcription inverse en chaîne par polymérase (RT-PCR) a été utilisée pour identifier les informations génétiques de l'arbovirus transmis par les moustiques dans les homogénats. Des échantillons positifs à ces gènes ont été inoculés dans la lignée (BHK-21) de cellules de rein de bébé hamster pour isoler le virus. Un taux d’infection minimal a été calculé et des analyses phylogénétiques ont été effectuées.
Résultats: Un total de 7 943 moustiques ont été collectés appartenant à six espèces de quatre genres: *Culex tritaeniorhynchus* représentait 73.08% (5 805/7 943), *Culex pipiens pallens* 24,75% (1 966/7 943), et les 3% restants (104/7 943) étaient composés de *Anopheles sinensis*, *Aedes vexans*, *Aedes dorsalis*, et *Armigeres subalbatus*. Seize groupes étaient porteurs du virus de l'encéphalite japonaise (VEJ) fondés sur la technologie d'amplification en chaîne par polymérase en temps réel (RT-PCR) utilisant des amorces spécifiques de génes prémembrane du virus de l'encéphalite japonaise (VEJ) . Les analyses phylogénétiques ont montré que tous les virus de l'encéphalité japonaise (VEJ)

appartenaient au génotype I; deux groupes se sont révélés positifs en utilisant des amorces de gènes du virus Getah. (VGETV) De plus, une souche du virus de l'encéphalité japonaise (VEJ) (SXYC1523) a été isolée des espèces *C. pipiens pallens.* Ces résultats révèlent que le taux d'infection minimum du virus de l'encéphalite japonaise (VEJ) dans les échantillons de moustiques collectés dans les cours des agriculteurs locaux avec des exploitations porcines était de 7,39 / 1 000; celui des fermes porcines était de 2,68 / 1 000; tandis que le taux était nul dans les cours des agriculteurs dépourvus de fermes porcines.

Conclusions: Les régions à forte prévalence de l'encéphalite japonaise chez l'adulte étudiées dans cette étude sont encore des foyers d'épidémie naturelle causée par le virus de l'encéphalite japonaise (VEJ). L''installation d'exploitations porcines à proximité des habitations constitue un facteur de risque pour la prévalence de l'encéphalite japonaise (EJ) chez l'adulte. Pour éviter l'apparition de cas d'encéphalite japonaise chez l'adulte, il a été fortement recommandé que, hormis de continuer la mise en oeuvre du Programme élargi de vaccination chez les enfants, le gouvernement devrait inciter les agriculteurs locaux à cesser l'élevage de porcs dans leurs propres cours pour réduire la probabilité d'infection au virus de l'encéphalite japonaise (VEJ).

Translated from English version into French by veromarie, through

TRANSLATORS WITHOUT BORDERS

Выращивание свиней в приусадебных хозяйствах как потенциальный фактор риска распространения японского энцефалита среди взрослого населения в Китае

Xiaojie Ren (Сярцзя Рен), Shihong Fu (Шийхун Фу), Peifang Dai (Пейфан Даи), Huanyu Wang (Хуаню Ван), Yuanyuan Li (Юаньюань Ли), Xiaolong Li (Сяолун Ли), Wenwen Lei (Вэньвэнь Лэй), Xiaoyan Gao (Сяоянь Гао), Ying He (Ин Хэ), Zhi Lv (Чжи Люй), Jingxia Cheng (Цзинся Чен), Guiqin Wang (Гуйцинь Ван), Guodong Liang (Годунь Лян)

Аннотация

Исходная информация. Рост заболеваемости взрослого населения Китая, в частности, северных районов страны, японским энцефалитом (ЯЭ) вызывает все более серьезные опасения. Для выявления связи между активностью комаров, вирусом японского энцефалита (ВЯЭ) и заболеваемостью ЯЭ среди взрослого населения авторы провели на юге провинции Шаньси эпидемиологическое исследование.

Методика. На свинофермах и в приусадебных хозяйствах фермеров провинции Шаньси были собраны образцы комаров. Полученная из образцов биомасса была гомогенизирована и подвергнута центрифугированию. Для выявления наличия в гомогенизированной биомассе генов переносимого комарами арбовируса применялся метод полимеразной цепной реакции с обратной транскрипцией (ОТ-ПЦР). Для выделения вируса образцы, давшие положительную реакцию, высевались на культуру линии клеток почки детеныша хомячка (ВНК-21). Был

рассчитан минимальный уровень зараженности, были проведены филогенетические анализы.
Результаты. Было собрано 7 943 образцов комаров, принадлежащих к четырем родам: *Culex tritaeniorhynchus* (73,08%, 5 805/7 943), *Culex pipiens pallens* (24,75%, 1 966/7 943), плюс 3% (104/7 943) пришлось на *Anopheles sinensis*, *Aedes vexans*, *Aedes dorsalis* и *Armigeres subalbatus*. По результатам ОТ-ПЦР с использованием предмембранных праймеров с генами ВЯЭ было определено шестнадцать водоемов, образцы из которых дали положительную реакцию. Филогенетический анализ показал, что все образцы ВЯЭ принадлежали к генотипу I; два водоема дали положительную реакцию при использовании праймеров с генами GETV. Кроме того, один штамм ВЯЭ (SXYC1523) был выделен из образцов *C. pipiens pallens*. Полученные результаты показывают, что минимальная зараженность ВЯЭ образцов, собранных в приусадебных хозяйствах фермеров, где есть свинарники, составила 7,39/1 000, для свиноферм этот показатель составил 2,68/1 000, а для приусадебных хозяйств, где свинарников нет, он был равен нулю.
Выводы. Районы, где проводилось исследование, характеризуются высоким распространением ЯЭ среди взрослого населения; источником угрозы являются природные факторы. Наличие свинарников вблизи жилищ представляет собой потенциальный фактор риска и способствует заболеваемости взрослого населения японским энцефалитом. Для предотвращения заражения взрослого населения ЯЭ на местном уровне было рекомендовано, чтобы, наряду с дальнейшей реализацией расширенной программы иммунизации детей, в целях снижения риска заболеваемости ЯЭ правительственные органы в срочном порядке потребовали от местных фермеров отказа от выращивания свиней в приусадебных хозяйствах.

Translated from English version into Russian by Alexander Poddubnyy, through

Las pocilgas cerca de viviendas como un factor de riesgo potencial para la prevalencia de encefalitis japonesa de adultos en China

Xiaojie Ren, Shihong Fu,P eifang Dai, Huanyu Wang, Yuanyuan Li, Xiaolong Li, Wenwen Lei, Xiaoyan Gao, Ying He, Zhi Lv, Jingxia Cheng, Guiqin Wang, Guodong Liang

Resumen

Antecedentes: La tendencia al aumento de los casos de adultos con encefalitis japonesa (EJ) en China, particularmente en el norte de China, se ha convertido en un problema importante de salud pública. Hemos llevado a cabo una investigación epidemiológica en el sur de la provincia de Shanxi para examinar las relaciones entre los mosquitos, el virus de la encefalitis japonesa (VEJ), y casos de EJ en adultos.
Métodos: Se recolectaron especímenes de mosquitos en los patios de los hogares de agricultores y granjas de cerdos en la provincia de Shanxi. Los mosquitos se combinaron, se homogeneizaron, y se

centrifugaron. Se utilizó la reacción en cadena de transcripción inversa de la polimerasa (RT-PCR) para detectar genes de arbovirus transmitidos por mosquitos en homogeneizados. Los especímenes positivos para estos genes fueron inoculados en la línea celular del riñón de un hámster bebé (BHK-21) para aislar el virus. Se calculó el índice de infección mínima y se realizaron análisis filogenéticos.

Resultados: Se recolectaron un total de 7 943 mosquitos pertenecientes a seis especies en cuatro géneros; *Culex tritaeniorhynchus* representó el 73.08% (5 805/7 943), *Culex pipiens pallens* el 24.75% (1 966/7 943), y el restante 3% (104/7 943) consistió de *Anopheles sinensis*, *Aedes vexans*, *Aedes dorsalis*, y *Armigeres subalbatus*. 16 muestras eran positivas para el VEJ basadas en RT-PCR usando partidores de genes anidados en premembranas del VEJ. Los análisis filogenéticos demostraron que todos los VEJ pertenecían al genotipo I; dos muestras resultaban positivas usando partidores de genes Getah Virus (GETV). Además se aisló una cepa del VEJ (SXYC1523) de especímenes *C. Pipiens pallens* Estos resultados indican que la tasa mínima de infección del VEJ en especímenes de mosquitos recolectados en los patios de los hogares de los agricultores con pocilgas fue de 7.39/1 000; la tasa en granjas porcinas fue de 2.68/1 000; y la tasa en patios de agricultores sin pocilgas fue de cero.

Conclusiones: Las regiones de alta prevalencia de adultos con EJ investigados en este estudio siguen siendo el foco de epidemia natural del VEJ. Tener pocilgas cerca de viviendas es un factor potencial de riesgo que contribuye a la prevalencia de adultos con EJ. Para prevenir la ocurrencia de casos locales de adultos con EJ, se recomendó que, además de continuar a implementar el Programa Expandido de Inmunización para Niños, el gobierno debería instar a los agricultores locales a dejar de criar cerdos en sus patios para reducir la probabilidad de infección del VEJ.

Translated from English version into Spanish by Ana Elvia Carrasco-Bustillos, through

TRANSLATORS
WITHOUT BORDERS
